# Diter von Wettstein (Dietrich Holger Wettstein Ritter von Westersheim): September 20, 1929-April 13, 2017

**DOI:** 10.1007/s11120-017-0420-9

**Published:** 2017-07-25

**Authors:** J. Kenneth Hoober

**Affiliations:** Susavion Biosciences, Inc., Tempe, AZ 85281 USA

## Prelude

**Fig. 1:**
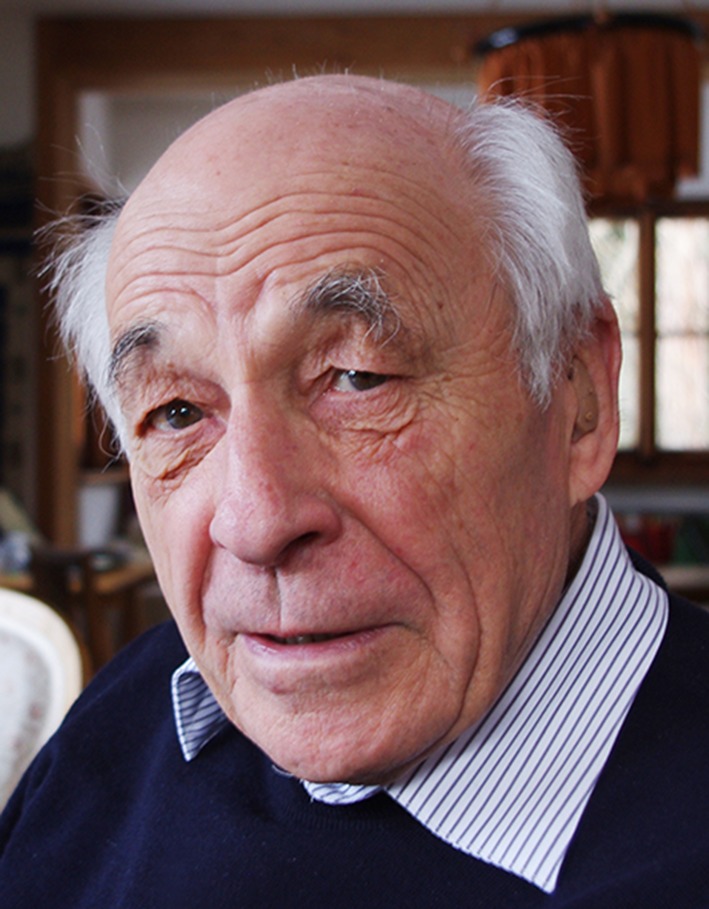
A portrait of Diter von Wettstein. Provided by Penny von Wettstein—Knowles

North of Copenhagen, outside of the small town of Hillerød, is Fredericksborg Castle, a seventeenth century, extraordinary building that houses the Danish Museum of National History. In the third floor attic, above the architectural glitter and portraits of the famous historical figures in Denmark, is a photo gallery of the more contemporary politicians, artists and entertainers. Photographs of only two scientists were in this gallery when I visited in 1987: Niels Bohr, who received the Nobel Prize in Physics in 1922, and Diter von Wettstein, which illustrates the stature that Diter enjoyed in Denmark (see Fig. 1 for a portrait; a selection of several photographs are in the Supplementary Material by Govindjee).

## Family background

Diter was born on September 20, 1929, in Göttingen, Germany, into an elite academic family. His grandfather (Richard von Wettstein Ritter von Westersheim) became Professor of Botany in Prague, The Czech Republic, and was Director of the Botanical Garden there. His grandmother (Adele) was an artist and studied painting with Gustav Klimt. His father (Friedrich “Fritz” Wettstein Ritter von Westersheim) conducted research in genetics in Berlin with Carl Correns at his Institute, and carried out his seminal work on the genetics, polyploidy, cytoplasmic inheritance and developmental physiology of mosses. Diter’s mother (Else Therese Jesser von Wettstein) studied botany with his grandfather in Vienna, Austria, during World War I and then went for 2 years to Uppsala in Sweden to study algae. In 1924, his father became Professor of Botany and Genetics at the University of Göttingen, as well as Director of the Botanical Garden. In 1931 his father moved to the University of Munich, as Professor of Botany, and in 1934 accepted the position previously held by Professor Carl Correns at the Kaiser Wilhelm Institute (now Max Planck Institute) for Biology in Berlin, Germany.

## Early research and training

Diter’s early career was under the tutelage of scientific giants of the early twentieth century, with mentors such as Albert Frey-Wyssling, Jakob Seiler, Åke Gustafsson, Erwin Bünning and co-workers, such as Arne Tiselius. With Gustafsson, Diter began his work on mutagenesis of barley and the genetics of chlorophyll synthesis. With Tiselius, he developed skill in electron microscopy, with which he obtained the earliest micrographs of developing chloroplasts (von Wettstein 1959a, b).

In 1953 Diter received two doctoral degrees, one from the University of Tübingen (Germany) for research in Biology/Biochemistry and a second from the University of Stockholm (Sweden) in Genetics. He also received a DSc degree in 1957 from the University of Stockholm in Genetics. From 1957 to 1962 he was Associate Professor in Genetics at Stockholm. In 1958 Diter obtained a Rockefeller fellowship to carry out research and training in the USA, first with Frits Went at the California Institute of Technology, Pasadena, California, for 3 months, then in the summer at Cold Spring Harbor, New York, to learn phage and bacterial genetics, and finally to do research in the last part of the year at the Carnegie Institute of Washington at Stanford University with James H.C. Smith and C. Stacy French on chlorophyll (bio)synthesis in barley mutants.

## Teaching and research in Sweden, Denmark and US

Upon his return to the University of Stockholm, Diter developed the first phage and bacterial genetics course in Sweden modeled after the Cold Spring Harbor courses, for which he could only find moral support and a teaching laboratory at the Microbiology Institute of the Karolinska Institute (the Medical faculty of Stockholm University). Many years later, Lars Rutberg, Professor of Microbiology in Lund, commented to Diter that he had taken that first course at the Karolinska Institute and that “…most present day professors in molecular biology in Sweden took your course”.

In 1962 Diter became Professor of Genetics and Head of the Institute of Genetics at the University of Copenhagen, Denmark. He had a long-standing interest in the process of chromosome pairing, on which he worked with Mogens Westergaard and described it as a “fascination” (von Wettstein 2006). In 1972 he was invited to join the Carlsberg Laboratory as Head of the Department of Physiology, a position he held until retirement in 1996. His negotiations for the position at the Carlsberg Laboratory resulted in construction of an excellent building for research and a major expansion in staff under his guidance. He was also the Head of the Carlsberg Plant Breeding group. During this time (1966), he was also a Visiting Professor at the University of California, Davis, CA, USA, collaborating with the prominent plant lipid biochemist Paul Stumpf on the biosynthesis of chloroplast lipids in his barley mutants (Appelqvist et al. 1968a, b), and also at Washington State University, Pullman, WA, USA. After his “official” retirement in 1996, he continued at Washington State University as the R. A. Nilan Distinguished Professor in the Department of Crop and Soil Sciences, School of Molecular Biosciences and the Center for Integrated Biotechnology.

During his years in Sweden and Denmark, Diter collected close to 360 mutant strains of barley that had deficiencies in pigment biosynthesis. These mutations were characterized genetically to more than 105 loci, a major contribution that he made to the Nordic Gene Bank and the Carlsberg Laboratory (Simpson and von Wettstein 1992). The mutants were organized according to pigment phenotype into several groups: *xantha, albina, viridis, tigrina, zonata*, and *chlorina*. Diter was a pioneer in applying integrated genetic, biochemical and microscopic analyses of these mutants. Analysis of the specific chlorophyll precursor that accumulated in these mutants allowed many of them to be placed on the biosynthetic pathway (von Wettstein et al. 1974, 1995). Diter’s electron microscopic examination showed a number of mutants to be defective in the assembly of thylakoid membranes, at specific steps. He was one of the first to describe the structural changes in plastid development from the proplastid to the chloroplast stage (von Wettstein 1959a, b). His conclusion that thylakoid membranes arise from vesicles from the plastid envelope required nearly 35 years for confirmation (Hoober et al. 1991, 2007).

Diter’s publications had an enormous impact on the developing field of plant biology, as described by Christoph Benning, Director of the Michigan State University—Department of Energy Plant Research Laboratory: “Diter von Wettstein inspired me throughout my career as he had brilliantly applied a combination of approaches, based not only on Genetics and Biochemistry, but on Cell Biology, to fundamental plant biological problems. His papers, for which I have numerous original reprints, in particular von Wettstein (1959b), guided my thinking about the formation of the thylakoid membrane, a fundamental process that is still not fully understood.”

Diter had the outstanding ability to attract excellent scientists to his laboratory. The Carlsberg Laboratory, in particular, became a *mecca* for many students, postdoctoral fellows and visiting scientists. His scientific interests were broad and he had a large number of collaborators that he supported in addition to those in his group. A major achievement by Diter and his team was elucidation of the initial steps in the pathway of chlorophyll biosynthesis. After Paul Castelfranco’s lab showed that 5-aminolevulinate, the first committed precursor of chlorophyll biosynthesis, is made intact from a 5-carbon precursor, Diter’s group identified a tRNA involved as glutamyl-tRNA (Kannangara et al. 1988; von Wettstein et al. 1995). They established that glutamate 1-semialdehyde was the product of reduction of glutamyl-tRNA, and revealed the mechanism of the enzymatic conversion of this interesting intermediate to 5-aminolevulinate by glutamate 1-semialdehyde aminotransferase (Grimm et al. 1992). The full story was published in a review in The Plant Cell (von Wettstein et al. 1995).

As the technique of gene mapping matured, Diter returned to his mutants and was successful in cloning several genes in the biosynthetic pathway of chlorophyll (Hansson et al. 1998). The convergence of sequencing the barley genome, development of microarray technologies, and identification of genes for chlorophyll biosynthesis in photosynthetic bacteria facilitated the cloning of barley genes (Zakhrabekova et al. 2002). In particular, Diter’s research group identified genes that are involved in the cyclase reaction that generates the isocyclic fifth ring and in the Mg chelatase step, needed to make the chlorophyll molecule (Rzeznicka et al. 2005). One of Diter’s coworkers remarked that “Diter is really good at picking problems that are solvable.” His wife, Penny von Wettstein—Knowles, frequently told people that *“*Diter had a Midas touch when it came to projects. If he said an idea was worth working on, it always was!” After identifying a problem, he would promise a solution if the agency would provide the funds for his basic research. It also did not hurt that the Carlsberg Laboratory was underwritten by the drinkers of copious amounts of Carlsberg beer!

## Research for the benefit of all of us: from the basics to the applied

The genius of his work was the direct coupling of question and answer. Diter engaged himself in a line of applied research to enhance the quality of barley for beer and animal and chicken feed. During malting, starch degradation by amylase is often incomplete because the heat destroys the β-glucanase that must depolymerize the aleurone layer surrounding the endosperm. A hybrid, codon-optimized, heat-tolerant β-glucanase was generated from a *Bacillus* species that when expressed in transgenic barley survived the malting process and increased the efficiency during making beer (Jensen et al. 1998). In addition, Diter identified genes involved in the synthesis of pro-anthocyanidins and, by breeding of mutants, he obtained strains that lacked these tannins (von Wettstein 2007). The beer made from these strains was clear without the need to remove haze by chemical treatments. Diter saw this as an interesting scientific question that he thought he could solve. In a major advance for agriculture, his group showed that a transgenic strain of barley, which expresses the β-glucanase, is much more easily digested by chickens (von Wettstein et al. 2007). This accomplishment allowed chickens to thrive on a diet essentially free of corn. He has also genetically engineered barley to achieve strains that are resistant to stem rust fungus, which improved agricultural yields (Horvath et al. 2003; Nirmala et al. 2011). He received nearly $2 million from the National Institute of Health (NIH), USA, and the Washington Life Science Discovery Fund to develop gluten-free strains of wheat to allow people with celiac disorder to eat food containing wheat flour (Osorio et al. 2012). His team generated strains in which 70 to 80% of the glutens, the seed’s storage proteins that cause celiac disease, were successfully removed. This achievement was described in an excellent review (Rustgi et al. 2014). *Diter’s research group was immensely productive until the end of his life. He deserves his place next to Niels Bohr*.

## Honors

Diter contributed over 300 papers on genetics, plant breeding, chloroplast developmental physiology and cell biology to science publications. He was elected to the US National Academy of Sciences in 1981 as a Foreign Associate in Plant Biology. Overall, he was a member of about a dozen academies of science around the World, including the European Molecular Biology Organization (EMBO), Academia Europaea, Royal Danish Academy of Sciences, Royal Swedish Academy of Sciences, Royal Belgian Academy of Sciences, Academia Leopoldina, Swedish Seed Association and received a distinguished decoration for rendering outstanding service to the Republic of Austria by its President. He organized many conferences and gave innumerable lectures around the world. Diter received the 4th Lifetime Achievement Award of the Rebeiz Foundation in 2010 (http://www.vlpbp.org/ltaawardvonwettsteinceremony093010a.html; others before him were: Andy Benson; Paul Castelfranco; and Govindjee; see testimonials and photographs in Supplementary Material by Govindjee).

## Personal life

Diter was married to Penny von Wettstein-Knowles, an outstanding biochemist in the Department of Genetics at the University of Copenhagen, whom he met at the University of California-Davis. Along with Penny and their daughters, Heidi and Kim, Diter loved the Austrian Alps, where they have a chalet for hiking in the summer and skiing in the winter. A few years ago he endured hip replacement to allow him to continue hiking. He had a full and prosperous life.

## Electronic supplementary material

Below is the link to the electronic supplementary material.

Supplementary material 1 (PDF 8746 KB)
