# Supplementary material for: Diter von Wettstein (Dietrich Holger Wettstein Ritter von Westersheim): September 20, 1929-April 13, 2017
Source: Photosynth Res. 2017 Jul 25;134(1):107–10. doi: 10.1007/s11120-017-0420-9 (PMC5603627; doi:10.1007/s11120-017-0420-9)
Supplement: Supplementary file 1 — Supplementary material 1 (PDF 8746 KB) [file 11120_2017_420_MOESM1_ESM.pdf]

Supplementary Material

for

Diter von Wettstein (*Dietrich Holger Wettstein Ritter von Westersheim)*: September 20, 1929-April 13, 2017, by Kenneth Hoober

by

Govindjee

(e-mail: gov@illinois.edu)

Excerpted from a presentation (see the pdf file linked to Diter von Wettstein) at

http://www.life.illinois.edu/govindjee/honorsfrom.html

And see the ceremony for Diter Von Wettstein at the Rebeiz Foundation in 2010:

http://www.vlpbp.org/ltaawardvonwettsteinceremony093010a.html

The photograph of Diter and Govindjee shown at the end of this Supplementary Material was taken by Laurent Gasquet

Govindjee thanks David J. Simpson for several photographs used in this presentation

September 18, 2010, Champaign-Urbana,Illinois, USA

Celebration for Diter Von Wettstein

"Man är blek och glåmig i blasten, med den von-Wettsteinska kloroplasten"

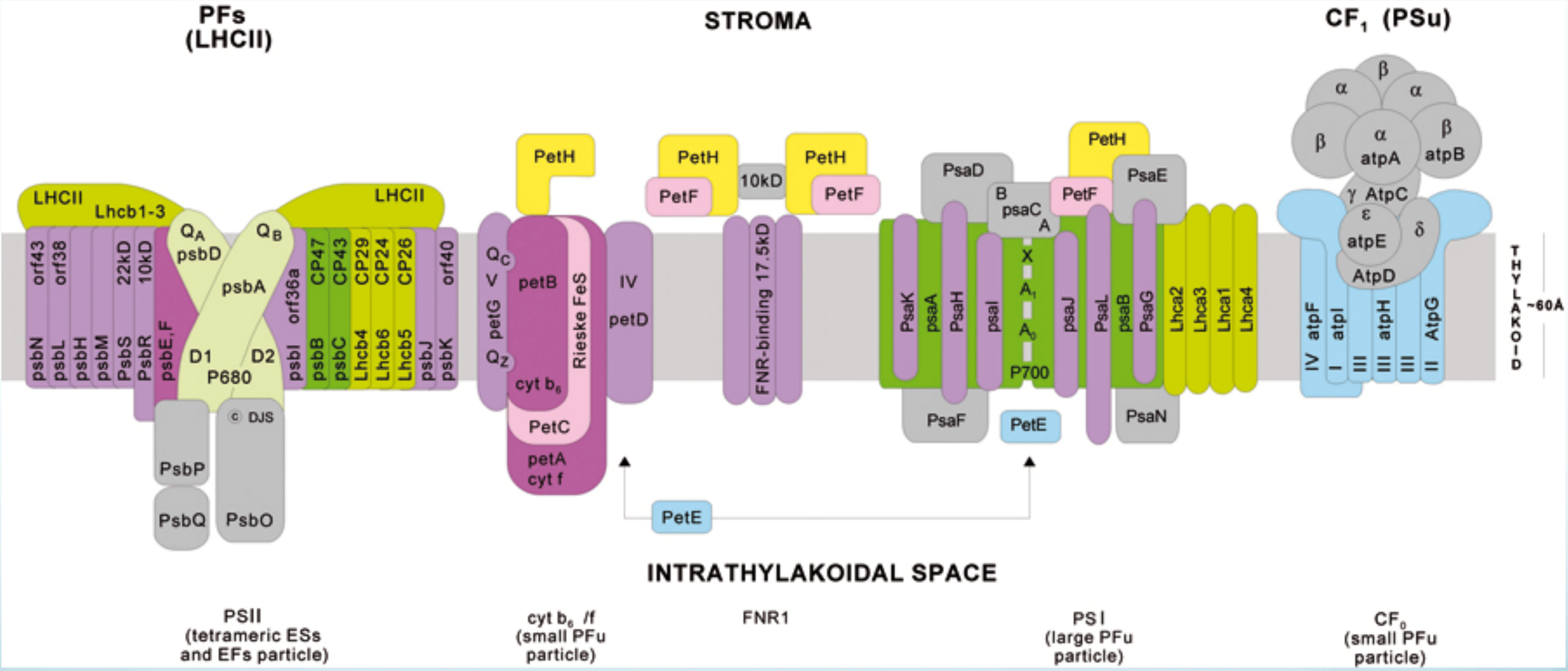

Happy Birthday to Diter in 2 days, September 20. And ....?

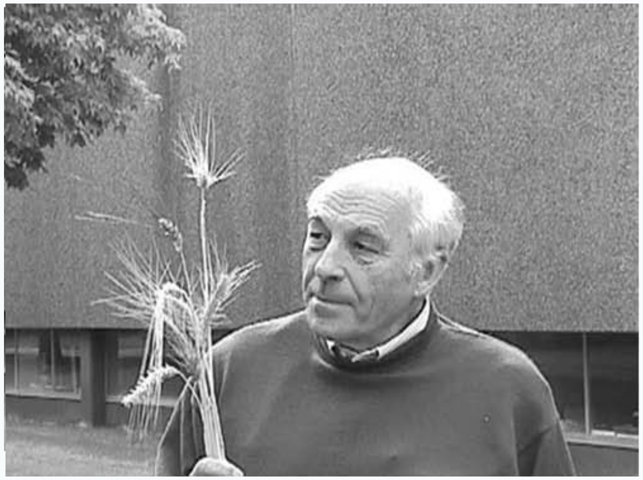

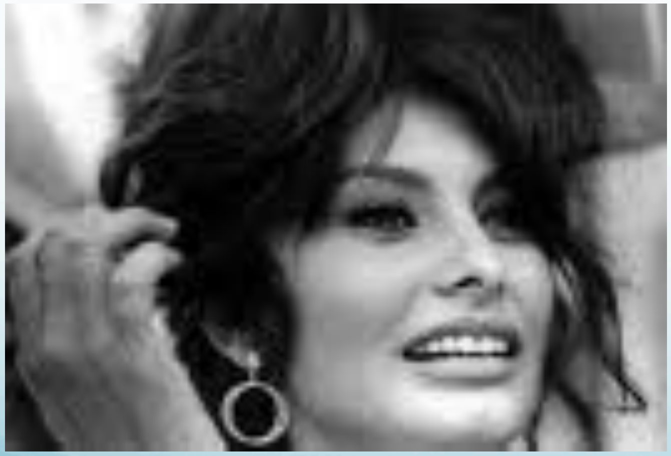

From: Barry Osmond (2006) Crassulacean acid metabolism; now and then. In (eds.) Esser U et al. (eds) Progress in Botany. Vol. 68, pp 1-32. Springer, Heidelberg.

"Previous title chapters in Progress in Botany, **from giants of European botanical research** in the latter half of the 20th Century, have explored fascinating and significant areas of plant science. **I am honored, and more than a little over-awed**, by the Editor's invitation to contribute in this context (and alarmed to discover that I am only, but precisely, a decade younger than the previous contributor!!)". Diter wrote the title chapter for 2005, and **the discovery of his sharing birthday was based on the Series editorial policy calling for publication of a brief CV**. ......[**Well, you can see who else shares his birthday**]

Andy Benson, last year's Awardee wrote

Andy Benson

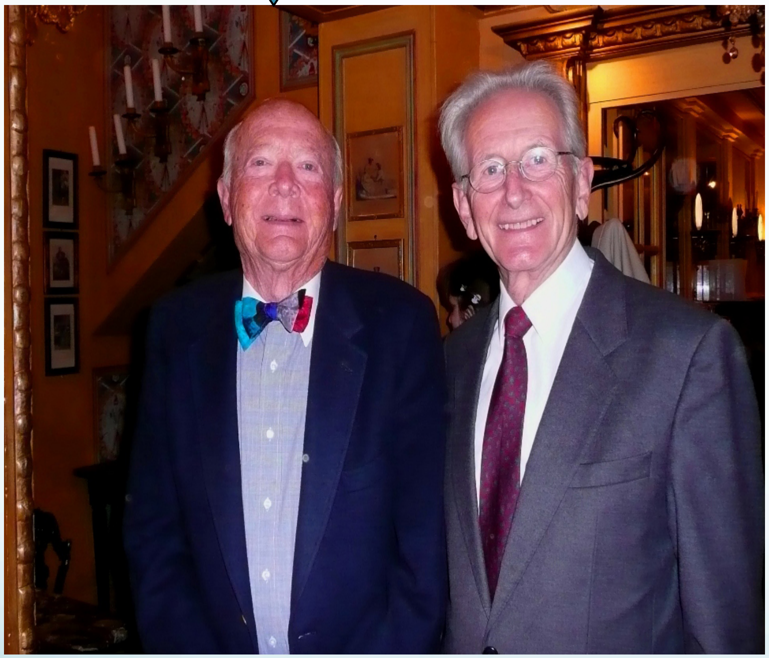

"I am pleased to learn that Diter von Wettstein has been selected for the prestigious Lifetime Award of the Rebiez Foundation. There could be no finer scientist in biological sciences than Diter. He has been a giant among giants in many areas of biology, from chemistry to genetics. Without self - assertion, he has ascended to leadership in many fields for many years."

When was it ?

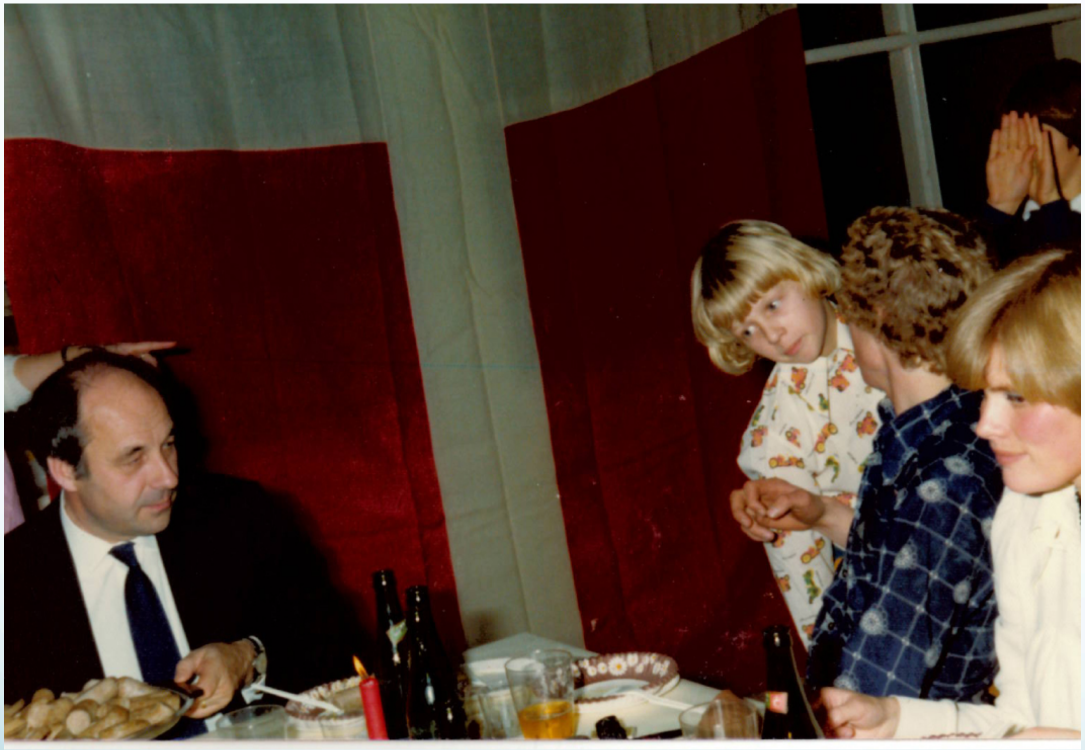

- **Diter seated at a table with beer bottles at a "harvest fest" at the Carslberg experimental farm.**
- **Quiz :Who are these onlookers?**

November, 1977

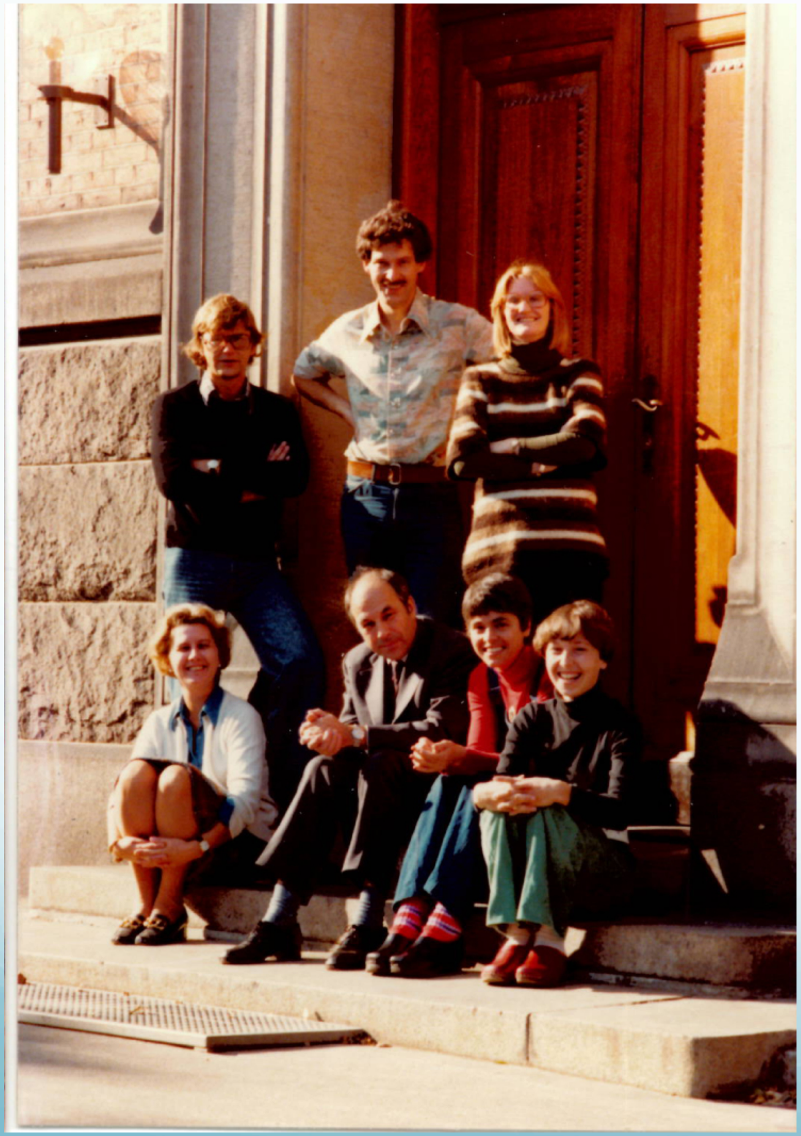

- **Diter is seated on the front stairs of the Carlsberg Lab**

Diter :Who are the others with you?

November, 1977

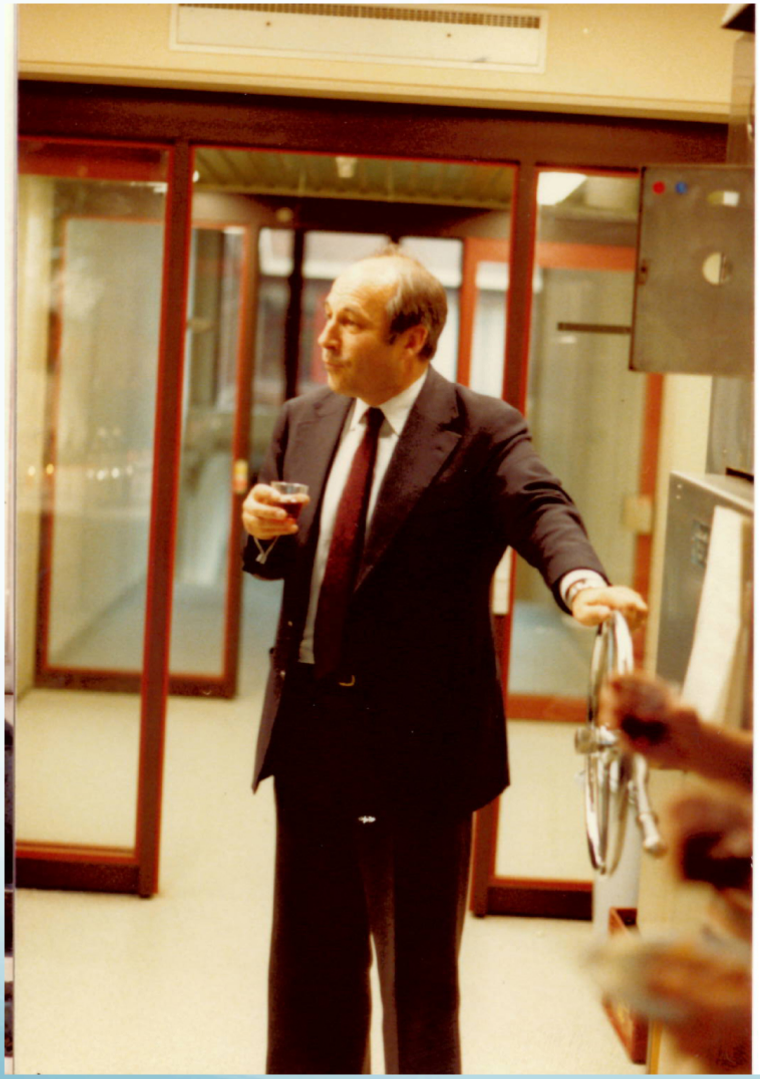

Diter is standing in his new laboratory

Diter: What are you really doing besides drinking wine, your favorite pastime?

October, 1978

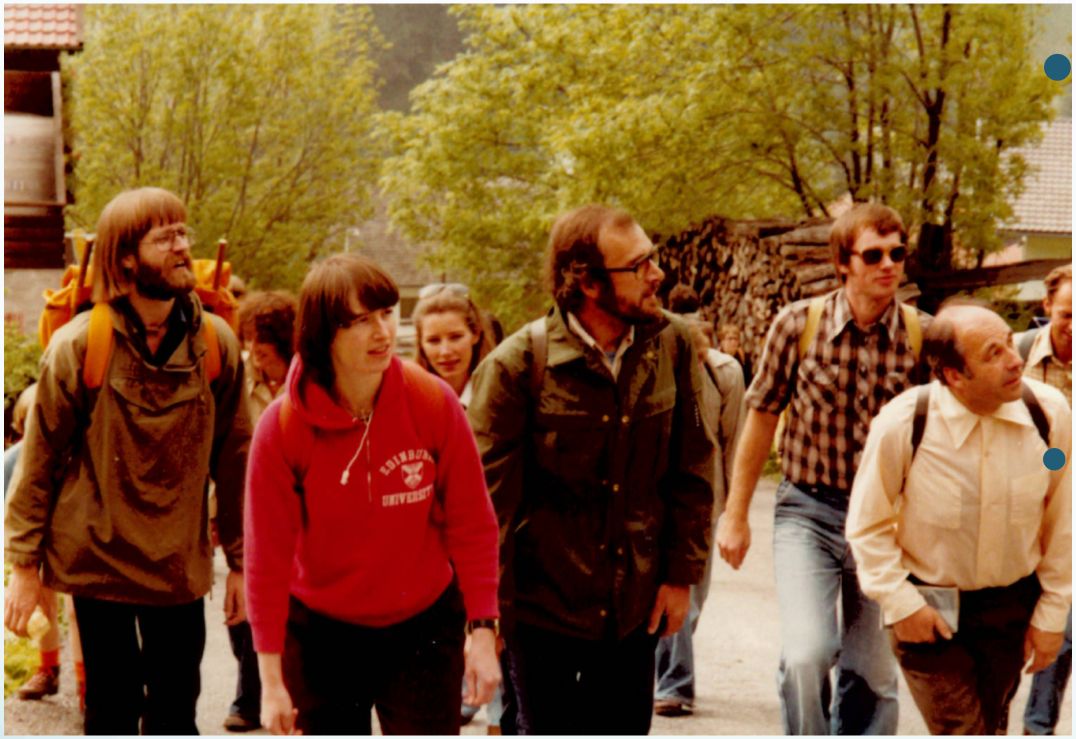

- **Diter with a group of scientists from the lab on a walk in the Danish forest.**
- **Diter: Who are the other scientists? And, which Danish forest is it?**

In Trins, Austria, Diter's family home

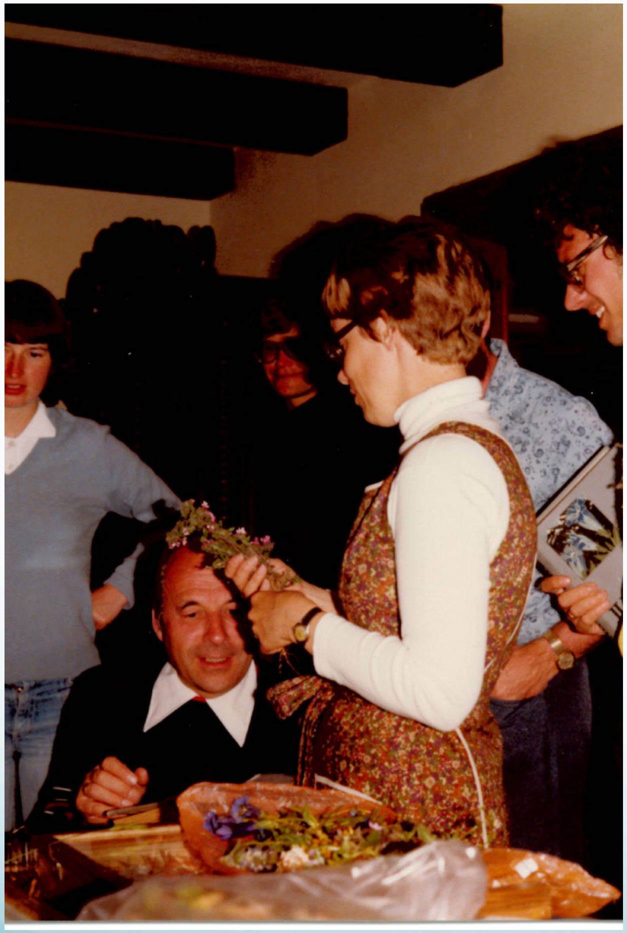

- **Diter is identifying some of the wildflowers collected on a 5-day visit to Austria on a botanical expedition, organized by the Lab**

- **Diter: What were the names of the wildflowers the others had collected? And who were those around you?**

In Trins, Austria…….

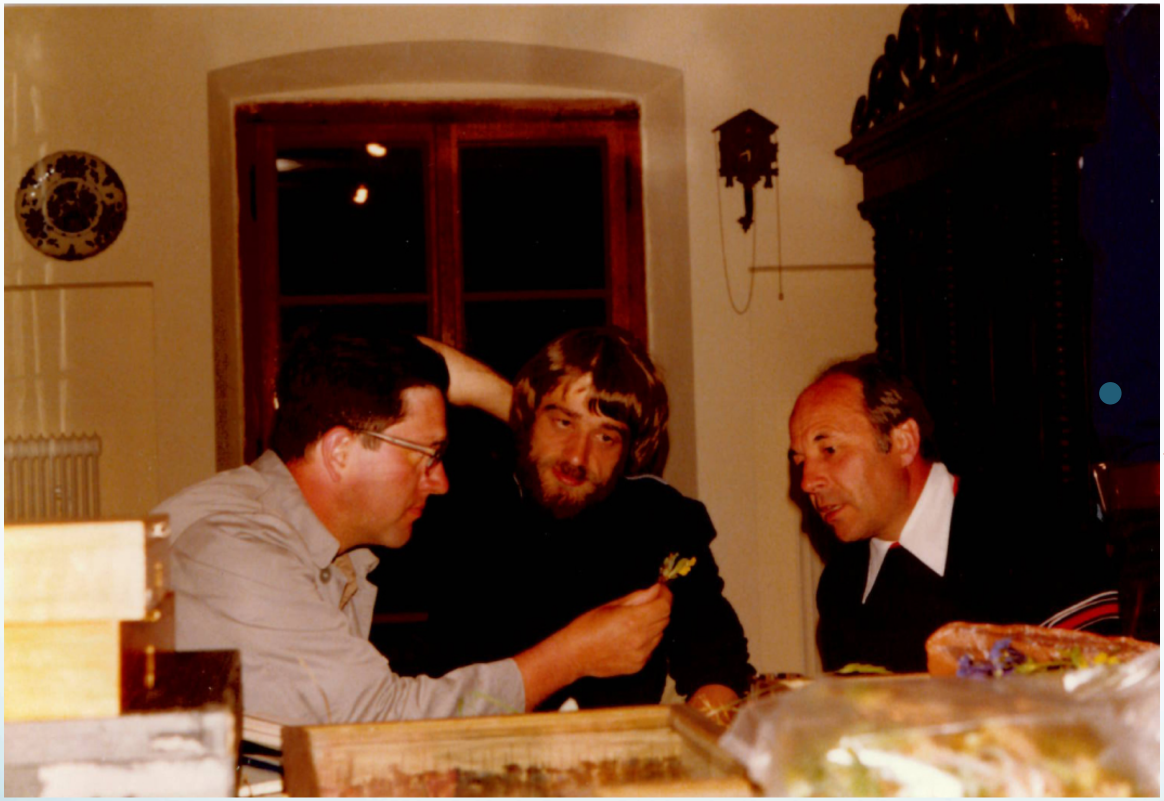

- **Diter: What wildflower was it? Who were the other two with you?**

Well, having fun…in Austria

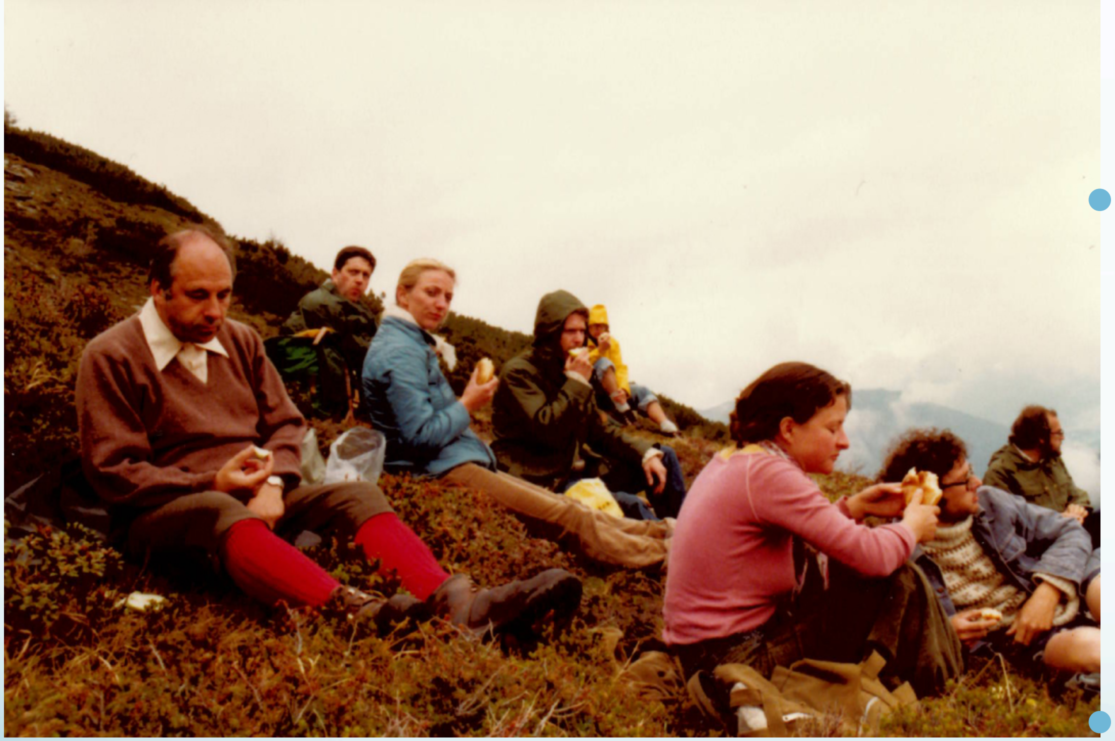

- **Diter is enjoying lunch on the mountainside.**
- **Diter:Who were the others?**

In Austria… on the top of a mountain

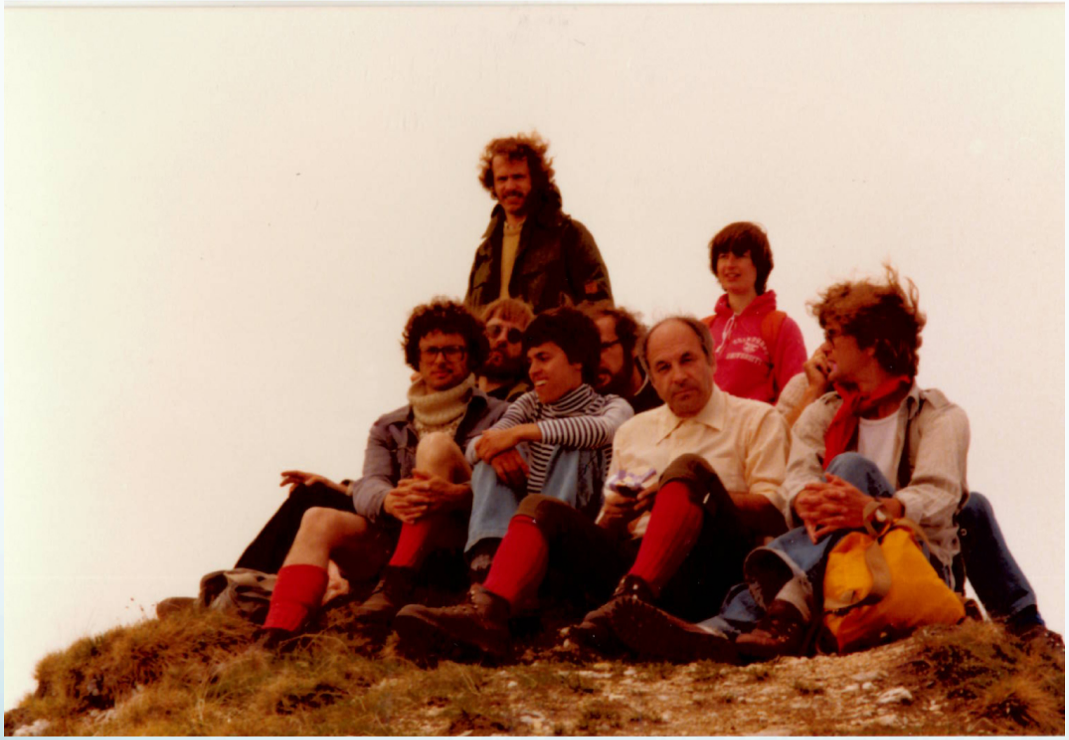

Diter: Which mountain
was it?

Who were the others?

In Austria..

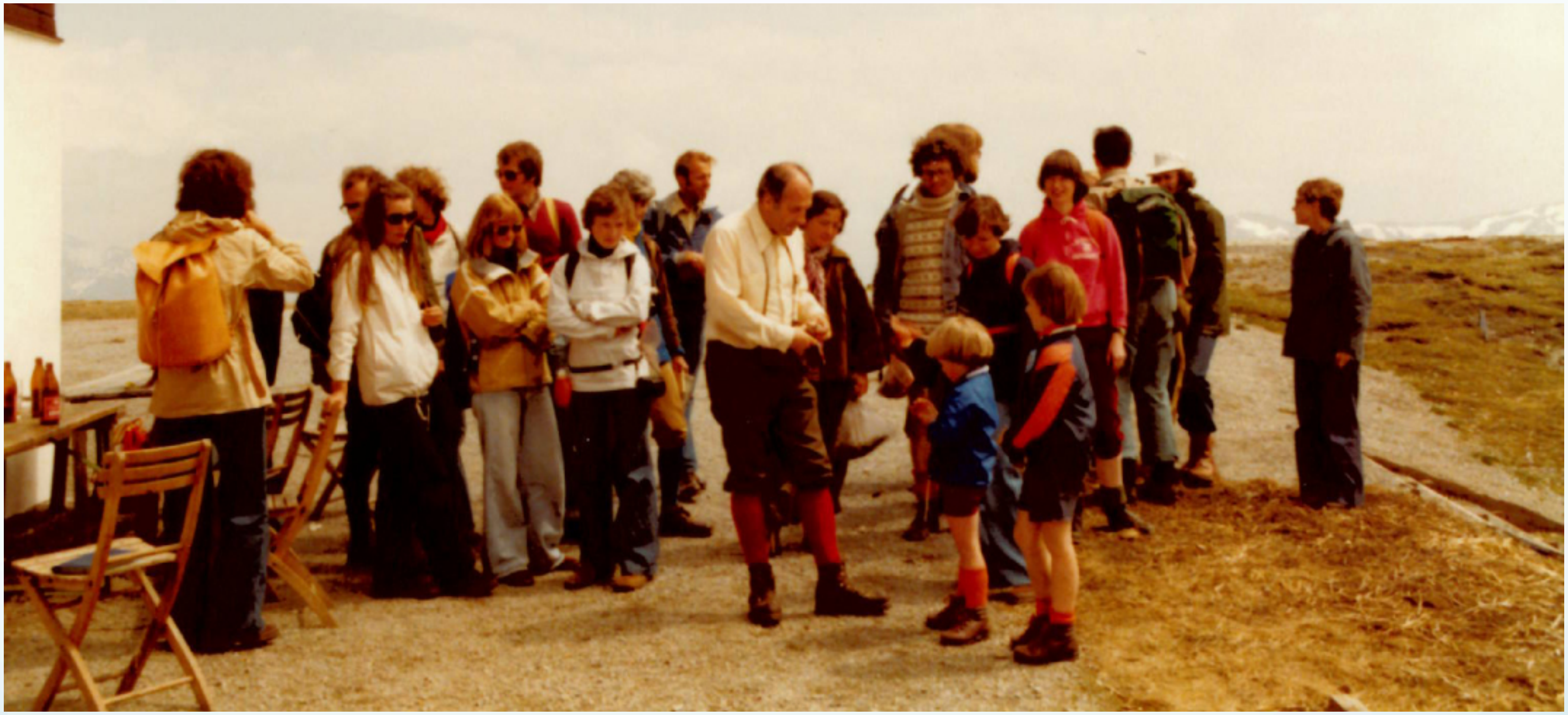

Diter with his daughters Kim and Heidi--
no more questions from Govindjee

Acknowledgment

- **Tino Rebeiz** for the opportunity to honor Diter
- Diter von Wettstein for providing me a copy of a letter and his notes on his ancestors
- Robert Clegg and Sanjay Govindjee for their help with the translation from German into English
- David Simpson for providing me photographs of Diter shown today
- Lars Olof Bjorn for the text, used in my slide 1, that was taken from a banner used by Diter's fans at a lecture given by Diter in Lund, Sweden, long time ago

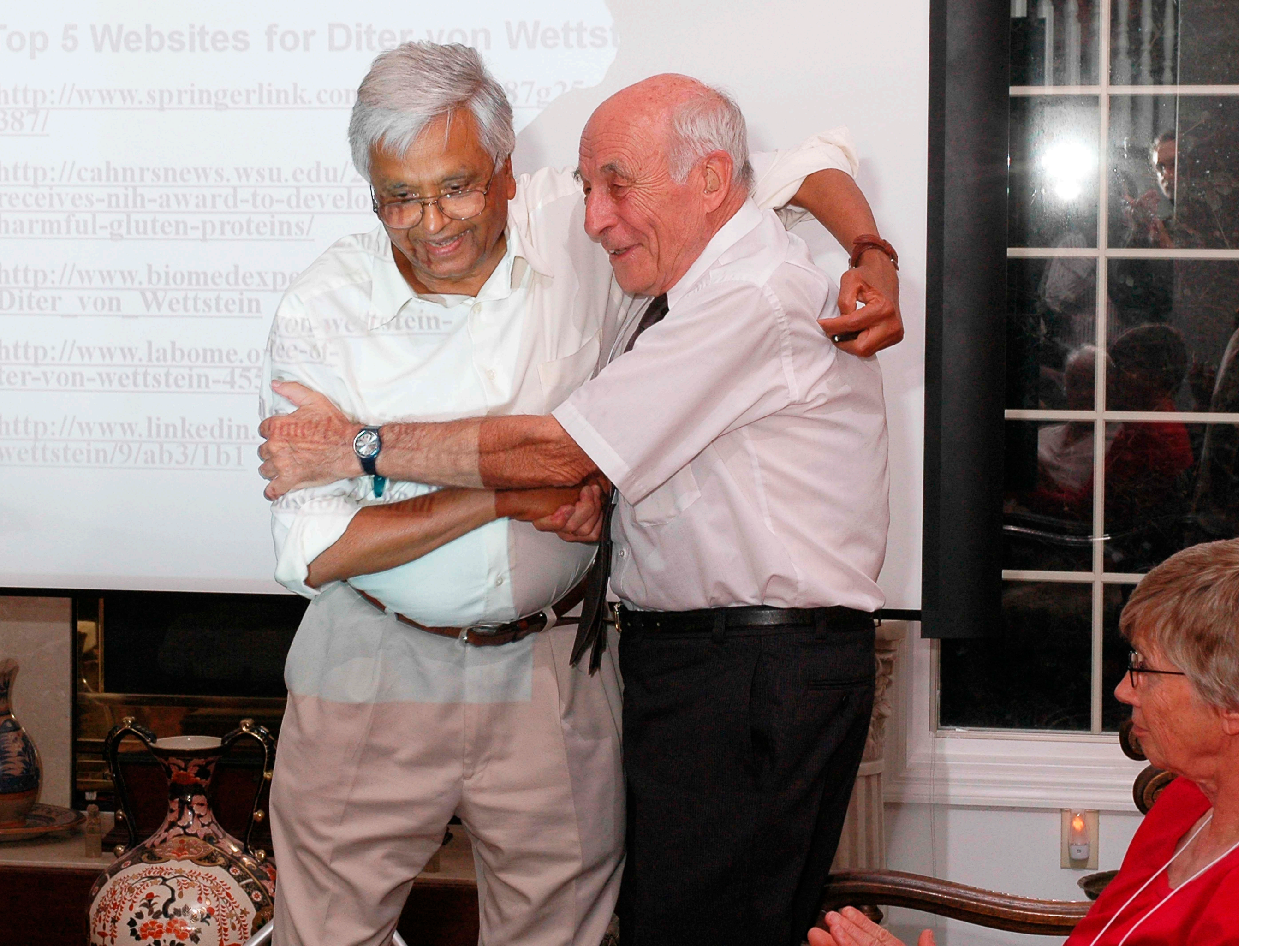
Top 5 Websites for Diter von Wettst
http://www.springerlink.com
harmful-gluten-proteins/
http://www.biomedexpe
Diter_von_Wettstein
http://www.labome.o
ter-von-wettstein-45
http://www.linkedin.
wettstein/9/ab3/1b1
